# Evidence for the importance of trophically transmitted parasites and predation at the lower latitudes of species ranges

**DOI:** 10.1098/rspb.2024.2039

**Published:** 2024-12-19

**Authors:** Anai Novoa, Ryan F. Hechinger

**Affiliations:** ^1^ Scripps Institution of Oceanography—Marine Biology Research Division, University of California San Diego, La Jolla, CA 92037, USA

**Keywords:** biogeography, distributional patterns, estuarine fish, latitudinal gradient, parasite diversity, parasite load

## Abstract

The lack of information concerning how parasitism maps onto host geographical distributions represents a striking gap in ecological knowledge. This knowledge gap limits our understanding of a wide range of phenomena, including the consequences of climate change-induced range shifts of both hosts and parasites. To help solve this problem, we created a predictive theoretical framework and quantified latitudinal variation in parasitism by animal and protozoan parasites throughout the entire contiguous geographical ranges of four estuarine fish species. To circumvent frequent limitations in data and to permit revealing of novel biogeographical patterns, we (i) quantified parasitism at the individual host level, (ii) quantified parasite species diversity and biomass load, and (iii) tracked functionally distinct parasitic consumer strategies. Parasite diversity always increased at lower latitudes, and this pattern was largely driven by parasites using trophic transmission. Furthermore, in three of four cases, the role of fish as predator- versus prey-host for trophically transmitted parasite (TTP) species increased at lower latitudes. Parasite diversity patterns followed predictions more consistently than did biomass load, indicating that increased predation at lower latitudes may decrease observed parasite biomass load. These findings suggest a particularly strong role for TTPs and predation in community structure and dynamics at lower latitudes.

## 1. Introduction

Many basic patterns concerning parasitism and host biogeography remain poorly documented or unknown [1–3]. This knowledge gap hinders our ability to understand the nature of species geographical distributions, given the importance of parasitism for hosts (reviewed in [4]) and even ecosystems ([5,6], reviewed by [7]). To address this gap, we theoretically and empirically examine how parasite community diversity and load vary among latitudes within the ranges of four host species.

Understanding how parasitism varies throughout host-species ranges would be useful for numerous reasons. One reason has to do with the growing number of species changing their distributions in response to climate change [8–10]. The large number of species ‘invading’ higher latitude communities has led to questions about how these new species will impact their new communities [11,12]. A major concern is that range-expanding species will bring their parasites with them, thereby exposing their new communities to novel parasites [13,14]. Partly countering this concern, but just as compelling, are basic reasoning and limited empirical data indicating that range-expanding hosts will often leave their parasites behind, resulting in novel ‘under-parasitized’ species in their new communities [15–17]. We could better predict the impacts of range shifts on parasitism and infectious disease if we had a basic understanding of how parasitism maps onto host geographical ranges. For instance, do host populations have different aggregate levels and types of parasites at range centres compared with range boundaries? Are there differences in parasitism at higher versus lower latitude range boundaries? Do host populations have lower levels and types of parasites in expanded compared with historical ranges? Such questions would clearly be best answered by having good data on most parasite species infecting host species throughout their ranges. Beyond informing climate change issues, answering these questions should provide a valuable insight for other aspects of basic and applied ecology, biogeography and evolutionary biology.

Despite the seemingly obvious utility of documenting how parasite communities and overall levels of parasitism vary within host-species geographical distributions, studies that provide data appropriate for quantifying such patterns appear to be quite rare [18]. Even studies focusing on a single parasite species or taxon throughout the range of a host are rare (e.g. [19–21]) and arguably suffer from inadequate sampling to accurately depict range-wide patterns of parasitism. In perhaps the outstanding exception, Blaylock *et al.* [18] conducted an extensive quantification of metazoan parasites of a fish (Pacific halibut, *Hippoglossus stenolepis*) from throughout much of the fish’s range in the northeastern Pacific. However, they had no samples from the southern third of the reported latitudinal range, where the fish is relatively rare. Yet, that area is also where important changes in parasitism might occur, including those setting the host’s lower latitude range boundary. Nevertheless, Blaylock *et al.* were still able to report a ‘weak and probably not biologically significant’ increase in parasite species richness at higher latitudes and a relatively strong increase in the total number of parasites per host at lower latitudes. Their study highlights the promise of revealing range-wide patterns of overall parasitism in hosts with appropriate sampling.

Another possible issue is that most previous parasite-community biogeographical work has focused on parasite species diversity and not on some direct metric of parasite load. Parasite diversity is useful but could benefit from some form of abundance information to possibly infer transmission dynamics and parasite impacts on host individuals. However, the typical currency for parasite abundance is the number of individual parasites. Using numbers of individual parasites in multi-species parasite communities can substantially misrepresent overall parasite load, particularly when different parasites vary greatly in body size. For this reason, the use of currencies such as aggregate biomass or estimated energy flux of parasites may provide a more adequate or complementary way to assess, for instance, the aggregate impact of parasites on host individuals [22–25] and how that might change with host biogeography.

Previous biogeographical research has also lacked consideration of the very distinct types of parasitic consumer strategies (*sensu* [26,27]). These consumer strategies—which can vary among life stages of any specific species—differ in fundamental ecological attributes, including how parasites impact host fitness. For example, a single ‘typical parasite’ *often* has little effect on its host, a single ‘parasitic castrator’ *always* reproductively destroys the host, and a single ‘parasitoid’ *always* kills the host. Also, ‘trophically transmitted parasites’ (TTPs) only transmit via predator–prey interactions and therefore provide unique information regarding host predator–prey interactions [26,28]. Recognizing this functional aspect of parasite diversity in biogeographical work may provide novel insight. Such insight could include the processes driving geographical ranges and inform our ability to forecast what parasites or functional groups of parasites are likely to move as hosts shift their geographical ranges. Indeed, there have been recent calls for biogeography to incorporate functional diversity to better represent biodiversity and better assess biogeographical patterns [3,29].

The above considerations led us to collect new data to begin to adequately address the following questions:

1. Does parasite diversity and aggregate load in individual hosts follow consistent geographical patterns throughout the host latitudinal range?
2. Can the same be said for the distinct types of functional parasitic consumer strategies?
3. Can we predict what those patterns might be, given basic biogeographical, ecological parasitology and epidemiological theory?

We quantified the latitudinal variation in parasitism by animal and protozoan parasites throughout the entire contiguous geographical ranges along the West Coast of North America of four estuarine fish species. One advantage in studying such coastal species is that conducting adequate sampling throughout their geographical ranges is relatively tractable as those ranges are essentially one-dimensional [30]. We quantified parasitism (1) at the single host (infracommunity) level, and (2) using diversity (species richness) and aggregate biomass load, while (3) considering the distinct parasitic consumer strategies. Furthermore, we conducted our work in a theoretical framework that we constructed pulling from biogeography, ecological parasitology and epidemiology to generate *a priori* and sometimes novel hypotheses and predictions (see below). The observed patterns of parasite diversity better met theoretical predictions than did parasite load and revealed a substantial increase in TTP diversity at the lower latitudes of each host species’ range.

### (a) Theoretical framework

Although little is known about how parasitism maps onto host geographical ranges, we formulated or compiled from the literature hypotheses and predictions using basic principles of biogeography, ecological parasitology and epidemiology. The hypotheses are not always novel or mutually exclusive, as multiple processes may operate simultaneously. We describe our predictions derived from the hypotheses below and summarize them in electronic supplementary material, table S1. The framework is broader than our current study—hence, we generate some hypotheses and predictions that we were unable to answer here (e.g. Hypothesis 1).

#### (i) Hypothesis 1 (H_1_)

Geographical gradients in parasite richness and load will follow latitudinal gradients in host density. Host population density is a central parameter in epidemiological models [31,32] that increases the likelihood that a parasite will transmit and persist in a host population. Thus, all else being equal, hosts occurring at high population densities should acquire more parasites and be more readily colonized by several parasite species than hosts living at lower population densities [33,34]. Thus, we predict that parasite diversity and load will each track geographical gradients in host density. We were unable to directly test this hypothesis because we did not quantify host density nor can we rely on the literature to strongly infer trends for the study species. However, we take two widely recognized distributional patterns that may characterize the study species and provide the specific prediction for those cases. Furthermore, we present a third case where a negative density-dependent relationship would be expected:

**H_1A_**: If host populations follow the ‘abundant centre hypothesis’ [35,36], characterized by a gradual decline in density towards range edges, then we predict that parasite richness and load will decrease towards the host’s range edges.

**H_1B_**: If host communities follow the typical ‘latitudinal diversity gradient’ (LDG) [37,38], wherein biodiversity increases from the poles to the tropics, densities of any specific host species may be lower or more fragmented at lower latitudes [39], driving a prediction that parasite richness and load will decrease towards the tropics.

**H_1C_**: In contrast, if parasite infective-stage production is decoupled from parasite transmission to a focal host (e.g. parasites with complex life cycles; see [40]), then higher local host densities can decrease *per capita* infection risk because the same number of parasites is divided among more available hosts [40,41]. If such ‘dilution’ conditions predominate, if host densities are greatest at higher latitudes, we would predict that parasite biomass load will increase towards the tropics.

#### (ii) Hypothesis 2 (H_2_)

Geographical gradients in parasite richness and load will follow host richness. To the extent that host diversity begets parasite diversity, and the greater surrounding diversity of free-living species at lower latitudes facilitates the transfer of parasites among species or fosters complex life cycle completion [42–44], we predict that *parasite richness and load* will increase at lower latitudes. While we did not directly quantify host richness, we *can* rely upon well documented patterns that multiple taxa [45], including estuarine fauna along the North American Pacific Coast [46], follow the general LDG.

#### (iii) Hypothesis 3 (H_3_)

Geographical gradients in non-host diversity can reduce transmission. If the greater biodiversity at lower latitudes involves increased richness of non-host free-living species, they can act as decoys or consumers of parasite infective stages [39,47,48] and drive a prediction that parasite biomass load and richness will decrease at lower latitudes.

#### (iv) Hypothesis 4 (H_4_)

Geographical gradients in predation can reduce parasite load. If predation rates are more intense at lower latitudes [49,50], then a decrease in parasite load may occur because high predation rates reduce the accumulation of parasite infections in otherwise longer-lived host individuals. Therefore, all else being equal, we predict that parasite load will decrease at lower latitudes. This relationship should particularly apply to non-TTPs.

#### (v) Hypothesis 5 (H_5_)

Geographical gradients in the intensity of predation will foster parasites that use trophic transmission. If predation is more intense at species’ lower latitudes [49,51], then a specific functional group of parasites, TTPs, should be favoured. These parasites exploit predator–prey relationships, being transmitted to predators that eat prey containing the TTPs (electronic supplementary material, figure S1).

**H_5A_**: If predation on a host is more intense and diverse at lower latitudes, we predict that the host will harbour a greater diversity, and possibly abundance, of TTP stages at lower latitudes. This hypothesis pertains to the host serving as the ‘prey host’ (which contains the TTPs) in trophic transmission (electronic supplementary material, figure S1a).

**H_5B_**: If a focal host species is itself a more intense predator at lower latitudes (as may occur because warm water can magnify metabolic demands and increase consumption rates [52,53], particularly of ectothermic species like fish), we predict that the role of the focal host as ‘predator host’ in trophic transmission (electronic supplementary material, figure S1b) will disproportionately increase at lower latitudes.

## 2. Methods

### (a) Host sampling

**Figure 1.**
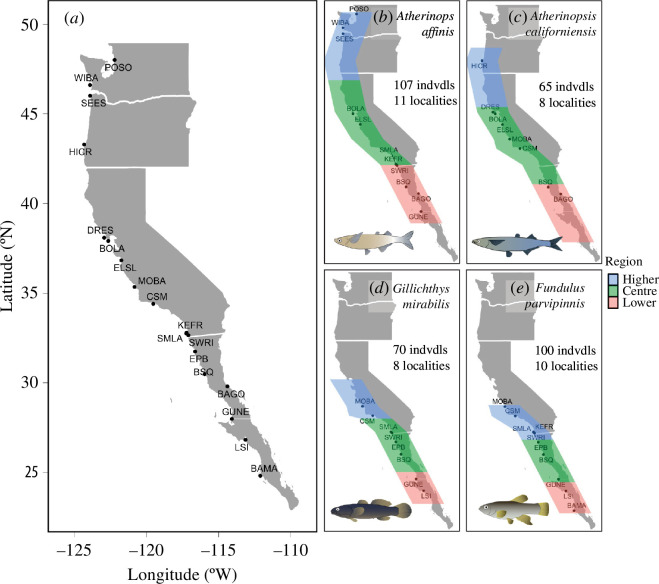
The collection localities, geographical range partitions and total sample sizes used for the four study species. (*a*) The full set of 18 collection localities along the West Coast of North America. Sample localities and range portioning for (*b*) *Atherinops affinis* (ATAF), (*c*) *Atherinopsis californiensis* (ATCA), (*d*) *Gillichthys mirabilis* (GIMI) and (*e*) *Fundulus parvipinnis* (FUPA). Locality codes are described in electronic supplementary material, table S2. Fish drawings by Azucena Rosales.

From June to September 2018 and August 2019, we used a two-pole seine (4′ high, 15′ wide, 1/8′ mesh) to collect four estuarine fish species from 18 localities throughout all or most of their known contiguous geographical ranges (*Atherinops affinis* (Ayres, 1860), Atherinopsidae, species code = ATAF, *n* = 107; *Atherinopsis californiensis* (Girard, 1854), Atherinopsidae, ATCA, *n* = 65; *Gillichthys mirabilis* (Cooper, 1864), Gobiidae, GIMI, *n* = 70; *Fundulus parvipinnis* (Girard, 1854), Fundulidae, FUPA, *n* = 100; figure 1; electronic supplementary material, table S2). We also sampled eight localities in the Gulf of California, but only found two of the fish species (ATAF and ATCA) at a single locality, Bahia Gonzaga. ATAF is reported from near Sooke Harbour, Vancouver Island, British Columbia to the Gulf of California; ATCA is reported from Yaquina Bay, Oregon to Bahia Magdalena, Baja California Sur and in the western and northeastern Gulf of California; GIMI is reported from Tomales Bay, California to the Gulf of California; FUPA is reported from Morro Bay, California to Baja California [54]. Immediately following capture, fish were individually bagged and frozen for later processing.

To help ensure sampling interspersion throughout each fish species’ latitudinal distribution, we sampled fish from multiple localities in each of three latitudinal partitions: the lowest 30%, the centre 40% and the highest 30% of each latitudinal range. We had an initial target of five localities per latitudinal partition, but the fish were not always found at each sampled locality.

Although our questions did not require extensive sampling at each locality (versus interspersed sampling among latitudes), we guided our effort by conducting sampling effort analyses of similar parasitological data that were previously obtained by the senior author (RH) and colleagues for three of the same host species from three different estuaries (data underlying [5]). Those analyses (based on the following total numbers—ATAF: 83, 50, 78; GIMI: 25, 13, 30; FUPA: 99, 116, 160) indicated that there was no bias in depicting parasite mean ‘infracommunity’ richness at even sample sizes <5 (A. Novoa, R. F. Hechinger, 2023 unpublished results). Hence, we set a target sample size of 10 individuals per locality for each host species to help ensure reasonable total sample sizes.

### (b) Parasite survey

We performed a comprehensive whole-body examination of each collected fish to detect a broad suite of metazoan and protozoan endo- and ectoparasites (and micropredators, see below). The fish was placed in a Petri dish, and total length, standard length, weight and sex were recorded. We examined the outer surface for ectoparasites under a dissecting microscope. Various parts of the fish were then removed, separately squashed between two glass plates and examined under the dissecting scope: scales from one side, all fins, gill arches from one side, separated visceral organs, the fillet from one side, the brain, one side of the head and one eye. Finally, the Petri dish water was inspected with transmitted light with a dissecting microscope. For bilateral organs, only one side (left or right side) was examined and parasite counts were doubled. During our examinations, we detected trematode metacercariae and adults, cestode metacestodes, monogeneans, nematode larvae and adults, acanthocephalan cystacanths and adults, copepods, cymothoid isopods, leeches, myxozoans and microsporidian cysts.

We assigned parasites and micropredators to morphospecies to broad taxonomic groups and identified them to the lowest taxonomic level possible using general guides for acanthocephalans [55], cestodes [56,57] and nematodes [58], supplemented with taxonomic literature from the Pacific Coast. We also assigned each to a different functional group, as described below.

### (c) Parasite metrics

We quantified parasitism at the individual host level (i.e. the ‘infracommunity level’ [59]) using parasite species diversity and parasite biomass density (as a measure of parasite load) expressed for the entire sampled parasite infracommunity and for its different functional partitions community (see below).

Parasite species diversity was expressed in its clearest form: species richness [60], the number of parasite species observed in a single host. By using individual hosts as sampling units, we avoided the issue of needing to account for the positive relationship between cumulative species richness and the number of hosts sampled [61].

We calculated parasite biomass density (g parasite tissue/g host) by dividing the total biomass load of parasites into each host’s directly weighed total ‘wet’ mass. We calculated the total biomass of parasites by multiplying their abundance in a host by species-specific estimates of individual parasite body or cell wet mass, which we obtained by either directly weighing individuals or estimating their mass by multiplying an estimate of their volume by a tissue density of 1.1 g ml^−1^ [5,25,62].

Concerning parasite functional groups, we used two different schemes. First, we separated species by their consumer strategy, *sensu* Lafferty & Kuris [26]; we encountered typical parasites, TTPs and pathogens. We rarely encountered species that may be appropriately categorized as micropredators (e.g. caligid copepods and leeches) instead of as typical parasites. Unlike parasites, micropredators feed on multiple hosts [26]. We included putative micropredators alongside our parasitic species because (1) they are considered by some people to be parasitic, (2) they are relatively small-bodied consumers (like parasites), (3) it was uncertain whether they do feed on multiple hosts (i.e. they may actually be typical parasites), and (4) they occurred in our samples.

The second functional grouping was by whether parasites use or do not use trophic transmission in their life cycle. Parasites using trophic transmission included TTP stages (those using the fish host as ‘prey host’) and typical parasites in the fish that we knew (based on the basic biology of the parasite’s taxon) had been trophically transmitted to the fish when it ate a prey item containing their earlier trophically transmitted stages (fish as ‘predator host’) (electronic supplementary material, figure S1).

### (d) Data analysis

**Figure 2.**
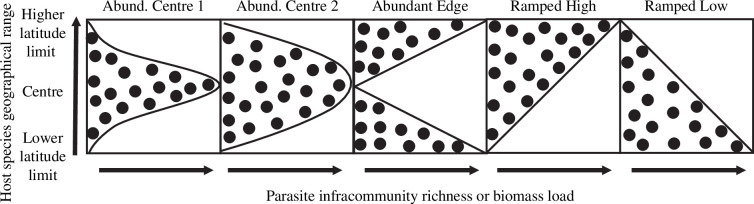
Five hypothetical distributional pattern ‘constraint curves’ describing parasite infracommunity richness and biomass load throughout a host species’ geographical range. These models were originally developed to fit patterns of local abundance within species ranges [30,63,64].

To evaluate how parasitism (e.g. parasite diversity and biomass density) maps onto fish species’ geographical ranges, we used two complementary approaches. The first approach was to use the method devised by Sagarin & Gaines [30] to examine geographical patterns of species abundance throughout the ranges of coastal species (as extended and implemented by Fenberg & Rivadeneira [63]). Specifically, we examined how well the upper bounds of the parasite data matched five competing models for how parasitism can change throughout host geographical ranges (figure 2). The five models represented different hypothesized ‘constraint curves’. Two of these curves model the ‘abundant centre’ hypothesis, assuming that maximum parasitism is reached at the range centre; however, one model uses a normal distribution and the other uses an inverse quadratic distribution to model a more gradual decline towards the edges. The ‘abundant edge’ model assumes the inverse pattern, with maximum parasitism observed towards the range edges and minimum values at the range centre. Finally, both ‘ramped high’ latitude and ‘ramped low’ latitude models assume that maximum parasitism declines from one range limit to the other and that intermediate values are reached at the range centre. To evaluate the fit of each model, we followed Fenberg & Rivadeneira’s [63] implementation in R [65], which uses randomization tests of the residual sum of squared deviations to generate *p*-values for the different models. We considered that a model adequately fits the observed data for *p* < 0.05. When multiple models were statistically favoured, we selected a top model using Akaike’s information criterion weight [63].

To provide a complementary and finer-grained understanding of the data, we also used generalized linear models (GLMs) to depict how mean infracommunity parasite richness and biomass density varied among latitudes (including the quadratic term). We used a log-link function and either a Poisson or negative binomial error structure as appropriate using the MASS package in R [66]. To express biomass density (a proportion) as a whole number suitable for Poisson or negative binomial regression, we multiplied it by whatever power of 10 permitted converting the value with the greatest number of decimal places to a whole number. In all but one case, GLM results paralleled those of the randomization tests.

To examine the relative change in the fishes’ role as a predator versus prey host for parasite species using trophic transmission (electronic supplementary material, figure S1), we used a GLM with a logit-link function and a binomial error distribution. Here, we used the parasite currencies that provided appropriate count data (e.g. individual parasites or micrograms of biomass).

We constructed our figures using the R package ggplot2 [67] and Microsoft Powerpoint. The reproducible code and the packages used are available in the online supplementary material.

## 3. Results

**Figure 3.**
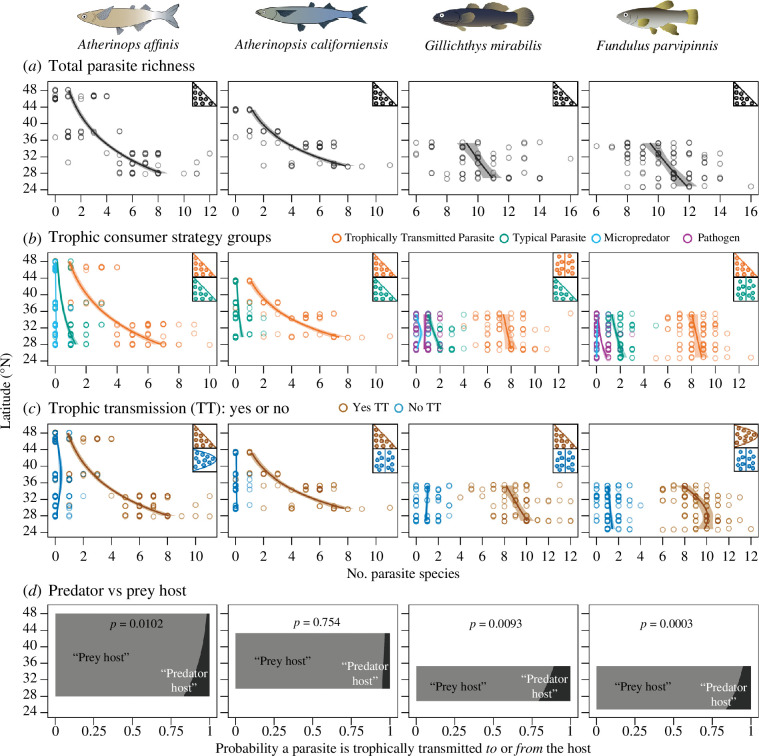
Latitudinal patterns of parasite infracommunity species richness throughout the ranges of four fish species: *Atherinops affinis* (ATAF), *Atherinopsis californiensis* (ATCA), *Gillichthys mirabilis* (GIMI) and *Fundulus parvipinnis* (FUPA): (*a*) of all parasite species, (*b*) within different parasitic consumer strategy groups, (*c*) of parasites with or without trophic transmission in their life cycle, and (*d*) the probability that a host serves as the prey- or the predator host for parasite species using trophic transmission. In (*a*)–(*c*), data points represent individual fish and are slightly vertically jittered and partly transparent to better reflect their density in the plot; regression lines represent predicted values for parasite richness from the top generalized linear models. Inset cartoons indicate the shape of the geographic distribution (from figure 2) of parasitism that was best fit and significant from the ‘constraint curve’ analyses (see electronic supplementary material, table S3). Fish drawings by Azucena Rosales.

As predicted from biogeographical and epidemiological theory, overall parasite diversity increased towards the lower latitudes of each fish species’ geographical range, consistent with the general LDG (figure 3*a*; electronic supplementary material, table S3). This increase in diversity at lower latitudes largely reflected the increased diversity of TTPs (figure 3*b*; electronic supplementary material, table S3). Decomposing parasite diversity by whether parasites have trophic transmission in their life cycle revealed that parasites with trophic transmission increasingly dominated parasite diversity at lower latitudes in all four fish species (although the ‘abundant centre’ pattern was also a significant fit for FUPA; electronic supplementary material, table S3). Additionally, the probability that a parasite species using trophic transmission was trophically transmitted to the fish as a predator host increased approximately 20% at lower latitudes for three out of four fish (figure 3*d*).

**Figure 4.**
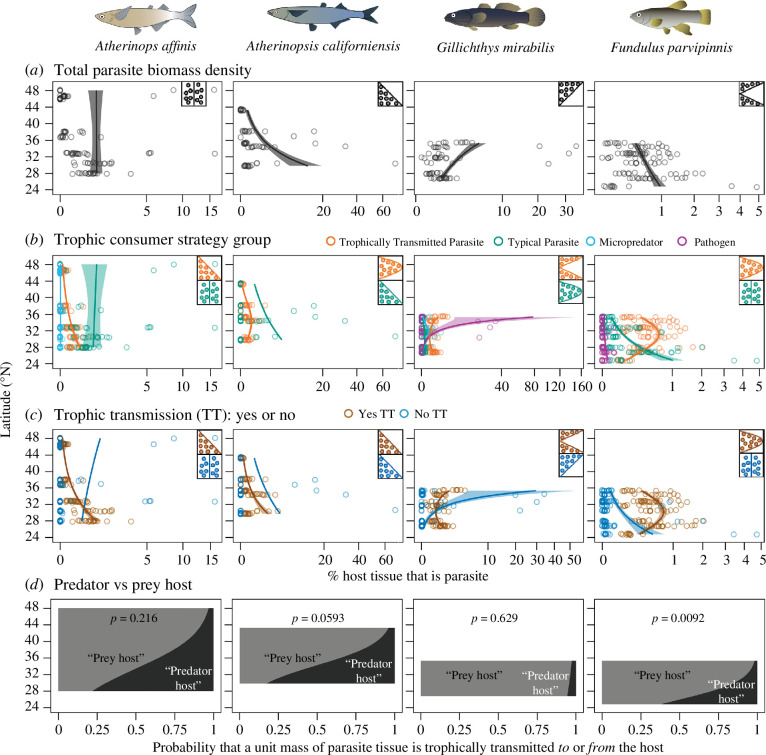
Latitudinal patterns of parasite infracommunity biomass load (density, g parasite/g total host weight) throughout the ranges of four fish species *Atherinops affinis* (ATAF), *Atherinopsis californiensis* (ATCA), *Gillichthys mirabilis* (GIMI) and *Fundulus parvipinnis* (FUPA) (*a*) of all parasite species, (*b*) within different parasitic consumer strategy groups, (*c*) of parasites with or without trophic transmission in their life cycle and (*d*) the probability that a host serves as the prey or the predator host for parasites using trophic transmission. In (*a*–*c*), data points represent individual fish and are slightly vertically jittered and partly transparent to better reflect their density in the plot; regression lines represent predicted values for parasite biomass density from the top generalized linear models. Inset cartoons indicate the shape of the geographical distribution (from figure 2) of parasitism that was the best fit and significant from the ‘constraint curve’ analyses (see electronic supplementary material, table S4). Fish drawings by Azucena Rosales.

Despite the relatively consistent trends of parasite diversity, parasite load (biomass density) was not consistently related with latitude. Biomass density increased at higher latitudes, lower latitudes or both range edges, depending on the host species and parasite consumer strategy (figure 4*a*–c, electronic supplementary material, table S4). The most repeatable trend was for the biomass density of typical parasites to increase at lower latitudes and TTPs to peak at the range centre (two of the four fish species). Furthermore, regardless of any changes among latitude in the loads of parasites using trophic transmission, for three of the four fish those loads were dominated by parasites trophically transmitted to the fish (fish as predator host) versus those transmitted trophically *from* the fish (fish as prey host) (figure 4*d*).

## 4. Discussion

In the context of theoretical expectations—formulated by drawing on basic principles of biogeography, parasite ecology and epidemiology—we examined latitudinal variation in parasitism throughout the entire contiguous geographical ranges of four estuarine fish species. To better reflect patterns of parasitism, we quantified parasitism (1) by numerous animal and protozoan parasites, (2) at the infracommunity (individual-host) level, (3) using both parasite diversity and biomass load, while (4) distinguishing distinct parasitic consumer strategies. These attributes of study design permitted novel biogeographical patterns of parasitism to be revealed, with implications for the role of parasitism throughout species ranges, the ecological consequences of shifting species ranges and our understanding of the distribution of parasite diversity throughout the globe.

### (a) Parasite species diversity followed the general latitudinal diversity gradient

Following the classical LDG, throughout each of the four species’ geographical ranges, parasite diversity in individual fish increased towards the tropics. This diversity trend was predicted under the ‘host diversity begets parasite diversity’ hypothesis, whereby the higher diversity of free-living species in an ecosystem can facilitate parasite diversity of any focal host species. Hence, one would expect relatively higher parasite diversity in host populations living in higher diversity areas, such as at the lower latitudes of their geographical range. This pattern is exactly what we found for each of the studied fish species, where parasite diversity increased by 25 to 800% at lower compared with higher latitudes.

Countering the clear trend that we observed, and the general expectation that parasite LDGs will parallel those of free-living species [68], there is no consensus in the literature for the empirical relationship between parasite species richness and latitude [3,68,69]. However, this lack of consensus may reflect the poor suitability of the data used for most evaluations of how the LDG applies to parasites. Such work is dominated by studies using information compiled from the literature on parasitism for many host species summarized at the host population or species level (e.g. [12,69–73]). Those data originate from different studies using different methods, different sampling efforts among hosts and geographical regions and different and limited assemblages of parasites. These issues can be difficult or even impossible to account for in literature compilation studies [1,2]. Hence, the appropriate data on parasite diversity may not yet exist for most host species, taxa or assemblages that would permit robust analyses concerning the generality of parasite LDGs.

To our knowledge, there may be suitable data in the literature to evaluate the parasite LDG within the range of only one host species: *Homo sapiens*. Humans are an exceptional case because the keen interest in human diseases has led to relatively good information on the geographical distribution of perhaps most eukaryotic parasites. Indeed, using a global dataset of human parasites, Guernier *et al.* [74] found that parasite richness of humans followed the classic LDG, with species richness increasing at lower (tropical) latitudes. This pattern parallels the general expectation for the LDG and what we report here for each of our studied fish species.

The latitudinal gradients for parasite diversity characterizing our work and the parasites of humans emphasize a problem of using the ‘host-filling’ assumption in literature-compilation studies [2]. Such studies assume, for want of data, that a parasite species reported from a host species at *any* part of its range will occur *everywhere* throughout the host’s range (e.g. see [71]. If the parasite LDGs that occur for humans and the four fish species studied here are general, it is easy to see how cross-species studies assuming no within-species gradients will weaken any global LDGs that might exist and otherwise influence global depictions of parasite diversity [2].

Hence, our findings underscore the necessity for more empirical work documenting parasite diversity throughout host species ranges to better assess the generality of increased parasite diversity at lower latitudes. In addition to possibly pointing the way to improved literature-compilation studies (e.g. perhaps such gradients could be incorporated to improve parasite diversity estimates derived from literature data), we will gain knowledge on what may be a pervasive biogeographical diversity pattern.

### (b) Parasite species diversity followed the latitudinal diversity gradient—implications for global climate change-induced range shifts

The observed decrease in parasite diversity at higher latitudes can inform predictions concerning climate change-induced range shifts and the spread of infectious diseases. Limited prior work suggests that—consistent with the general trend for invasive species [75,76]—such range-expanding species tend to bring a subset of the parasites that they host in their historical range [15–17]. It is reasonable to expect that parasites brought into expanded ranges will tend to be a subset of parasites found in the adjacent part of the historical range. Hence, with parasite LDGs like those documented here, we predict far fewer and different parasite species to follow the host into a higher-latitude expanded range than if we were simply to consider the suite of parasites known from throughout the entire range of the host.

### (c) The possible importance of host range size and range latitudinal position

Interestingly, we observed stronger trends in the two fish (ATAF and ATCA) that have broader geographical ranges compared with the other two species (GIMI and FUPA), which have narrower geographical and lower-latitude-positioned ranges. Parasite richness decreased about 10× more per degree of latitude for ATAF and ATCA than for GIMI and FUPA. Finding overall stronger LDG trends for ATAF and ATCA is consistent with the generality that LDGs are stronger when data span larger geographical ranges [60,77]. Perhaps, this trend also applies to parasite communities throughout host species ranges.

Of further interest, these two species with steeper parasite diversity gradients also substantially spanned two geographical provinces—the Californian (defined here as Point Conception to Bahia Magdalena) and the higher-latitude-positioned Oregonian (the lower-latitude tip of Vancouver Island to Point Conception). This might suggest the possibility that, rather than being continuous with latitude, diversity may increase stepwise in successive lower-latitude biogeographical provinces. However, inspecting the data indicates that this is not the case; there is a negative slope *within* each of the Californian and Oregonian provinces for these two species, and, despite the limited latitudinal range, one of those slopes was statistically significant (electronic supplementary material, table S5). Furthermore, although less steep, the two host species that were largely confined to the Californian Province also had parasite LDGs. Hence, our findings suggest that host species that are confined to single biogeographical provinces may experience parasite LDGs and that hosts that span multiple biogeographical provinces may experience relatively stronger shifts in parasite diversity throughout their ranges.

Despite having relatively small geographical ranges, GIMI and FUPA had 130–160% greater parasite diversity on average than the diversity characterizing the other two species throughout their entire ranges. This may seem to counter the general trend that species with larger geographical ranges have greater parasite diversity [72]. However, GIMI and FUPA are confined to the more low-latitude-positioned Californian province, and their relatively greater parasite diversity was greatly diminished when compared solely with populations of the other two species at the same latitudes (35–60% greater parasite diversity at 30°N). Therefore, the data suggest that absolute latitudinal position is a particularly important driver shaping parasite species richness and can counter any negative effects of having a small geographical range size.

### (d) Parasites using trophic transmission composed the bulk of parasite diversity and its increase at lower latitudes

We found that parasites using trophic transmission were particularly important components of parasite communities throughout the hosts’ geographical ranges. First, TTPs of the fish—those using the fish as prey host to transmit to predatory final hosts—composed the bulk of parasite diversity throughout the entire geographical ranges for each fish species. Second, TTPs drove most of the observed increase in parasite diversity at lower latitudes for three fish hosts (ATAF, ATCA and FUPA), with TTP diversity increasing by 25 to 800%. Further, there was no increase in diversity at lower latitudes for ‘typical parasites’ for three of the four host species when we removed the subset of typical parasites that had been trophically transmitted *to* the fish (i.e. parasites were TTPs in the prey host eaten by our focal fish host). Hence, all, or nearly all, of the lower latitude increase in parasite diversity was caused by the diversity of parasites trophically transmitting *to* or *from* the fish host.

We had predicted a greater diversity and perhaps abundance of parasites with trophic transmission at lower latitudes given the general expectation that predation intensity increases towards the tropics [49,50]. If the fish hosts serve as a prey or as predator at greater rates at lower latitudes, they may support a greater diversity of TTPs that benefit from that greater predation intensity. Under this hypothesis, TTPs are simply taking advantage of, or hitchhiking on, the greater intensity of predation. However, TTPs often do more than simply hitchhike on pre-existing predator–prey relationships; they frequently modify the behaviour and morphology of their hosts to increase the rate of predation on infected individuals [28,78,79]. Therefore, the absolute greater diversity of TTPs at lower latitudes may not only reflect such parasites taking advantage of a greater intensity of predation (hitch-hiking) but also that they may contribute to *increasing* the intensity of predation at lower latitudes.

Hence, our findings suggest that TTPs may play a disproportionately important role in their hosts’ ecology at the lower latitudes of their geographical ranges. Such a role could extend to influencing the hosts’ lower-latitude limits. This idea is consistent with the general idea that abiotic factors tend to be more limiting at higher latitudes, whereas biotic factors limit species’ distribution and abundance at lower latitudes [51,80,81].

### (e) Fish played a disproportionately greater role as predators in parasite trophic transmission at lower latitudes

We also found that fish disproportionately increased their role as a predator host for parasite species with trophic transmission at the lower latitudes of their geographical ranges. That is, in three out of four cases, moving towards the tropics, the chance that a parasite species with trophic transmission used the fish as predator host increased 2–20 times relative to the chance it used that fish as prey host. A parallel but even stronger pattern for parasite biomass load occurred, wherein a randomly sampled gram of parasite tissue was 7–80 times more likely to represent parasites trophically transmitted *to* the fish host versus trophically transmitted *from* the host. Fish serving a disproportionately greater role as predator hosts for parasites with trophic transmission at lower latitudes may reflect that the host predates at greater rates at lower latitudes. The latter should be expected, as the warmer temperatures at lower latitudes likely magnify the metabolic demands and increase consumption rates of these ectothermic predators [52,53].

The increase in the relative dominance for parasites using the fish as predator versus prey host in trophic transmission represents, to our knowledge, a novel biogeographical pattern. This pattern may have gone previously undetected given the general poor state of the data on parasites and latitude discussed above. But it may have also been undetected because parasite communities are rarely investigated while recognizing their distinct consumer strategies. Indeed, few biogeographical parasitological studies have incorporated any type of functional traits; among those that have, there is little consistency and only weak empirical support for latitudinal gradients of any parasite trait [3]. Given our findings and the fundamental nature of the discrete consumer strategies [26,27], we hope that more research will categorize parasites in this way to improve our understanding of the role of parasites in ecosystems and how that role may geographically vary.

### (f) Parasite biomass load does not always parallel latitudinal diversity trends, suggesting that epidemiological factors sometimes counter simple additive effects of increasing diversity

Despite the relatively repeatable trends for parasite diversity, parasite loads (biomass densities) were not consistently related with latitude among the four fish species. Parasite biomass density increased at the higher latitude, lower latitude or both range edges, depending on the host species and parasite consumer strategy. This lack of consistency was anticipated by our *a priori* expectations. For instance, although increased predation rates at lower latitudes may foster a greater diversity of TTPs (see above), modelling indicates that increased predation rates can reduce parasite load by shortening prey (host) lifespans and preventing the accumulation of parasites [82]. Furthermore, this dynamic is enhanced as parasites disproportionately increase predation rates on highly infected hosts, something implied by our data to be occurring with the greater diversity of TTPs at lower latitudes. Interestingly, the opposite is observed in predator hosts, wherein increasing predation rates tend to increase parasite prevalence [82,83]. This latter effect might explain why we saw the relative increase in the fish hosts serving as predator hosts versus prey hosts for parasites with trophic transmission. Hence, variability in the extent to which predation blocks the accumulation of parasite load might explain the lack of consistent geographical patterns concerning parasite load.

## 5. Conclusion

We conducted our work in the context of a theoretical framework and quantified the latitudinal variation in parasite diversity and load—while recognizing distinct parasitic consumer strategies—throughout the geographical ranges of four estuarine fish species. We found that parasite diversity patterns more consistently followed *a priori* predictions and hypotheses than did parasite load. Parasite diversity was lowest at the higher latitudes of each species range, suggesting that range-shifting species may be typically under-parasitized. In contrast, parasite diversity was greatest at the lower latitudes of each species range, consistent with the general LDG and the generality of host diversity begetting parasite diversity. Furthermore, consistent with the general expectation that predation is more intensive and diverse at lower latitudes, (1) the diversity of parasites that exploit predator–prey interactions was greater at the lower latitudes of each species range, and (2) the relative role of fish as predator versus prey host for TTPs was greater at lower latitudes in three out of four species, while (3) parasite biomass-load patterns were otherwise quite variable. Because our study hosts have overlapping geographical ranges, a shared habitat and shared phylogeny (e.g. they are all actinopterygian fish), the generality of these patterns and hypotheses remains to be tested. Yet, these findings do indicate that a greater diversity of parasites can impact host species at the lower latitude parts of their ranges and that TTPs and predation jointly play particularly important ecological roles towards the Equator.

## Data Availability

The data and code used in the analyses are available in the electronic supplementary material [84].
